# Heat and mass transfer for MHD peristaltic flow in a micropolar nanofluid: mathematical model with thermophysical features

**DOI:** 10.1038/s41598-022-26057-6

**Published:** 2022-12-13

**Authors:** A. M. Abd-Alla, S. M. Abo-Dahab, Esraa N. Thabet, M. A. Abdelhafez

**Affiliations:** 1 Department of Mathematics, Faculty of Science, Sohag University, Sohag, Egypt; 2 Department of Mathematics, Faculty of Science, South Valley University, Qena, Egypt

**Keywords:** Applied mathematics, Computational science, Nanoscience and technology

## Abstract

According to a survey of the literature, nanofluids are superior to traditional fluids at transferring heat. A detailed analysis of the models mentioned above is crucial since there are large gaps in the illumination of current solutions for improving heat transfer in nanomaterials. The ongoing investigation's purpose is to ascertain the tiny size gold particles drift in free with the heat and mass transfer, buoyancy forces, thermophoresis, and Brownian motion of a micropolar nanofluid being transported through a porous medium in an asymmetric channel with a uniform magnetic field using a long-wavelength and low Reynolds number approximation. The resulting dimensionless nonlinear governing equations have been numerically solved using a MATLAB software and the Runge–Kutta–Fehlberg integration scheme. Two comparisons with previously investigated problems are also made to confirm our findings, and an excellent concurrence is discovered. As a result, trustworthy results are being given. Numerical solutions are used to describe the effects of different thermal-fluidic parameters on velocity profiles, temperature, concentration, micropolar rotation, pressure gradient, shear stress, heat flux, and nanoparticle volume flux, etc. Tables, graphs, and bar charts are used to present and discuss numerical results that have been produced. A comparison of the resulting numerical solution to earlier literature also reveals a satisfactory level of agreement. Insight into real-world applications such nanofluidic, energy conservation, friction reduction, and power generation are provided by this work. Furthermore, the Brownian and thermophoresis parameters behave significantly differently in a concentration field. On the other hand, the study puts forward an important note that for peristaltic flow of a micropolar fluid with nanoparticles can be controlled by suitably adjusting the micropolar parameter, thermophoresis parameter, nanoparticle Grashof number, and Brownian motion parameter.

## Introduction

Nanofluids are liquid suspensions containing diluted nanoparticles, each of whose principal diameters is less than 100 nm. Even at low nanoparticle concentrations, they exhibit a significant improvement in their attributes. Understanding nanofluid behavior is a major focus of many papers on the subject since it paves the way for their employment in many industrial applications, nuclear reactors, transportation, electronics, biology, and food, where direct heat transfer enhancement is crucial. Nanofluids are smart fluids, where heat transfer can be reduced or improved at will, have also been testified. Nanofluids were became recognized as sophisticated heat transfer fluids in less than two decades. The properties of nanofluids, which are properly scattered nanoparticles, include high specific surfaces, a larger surface area for heat transfer between particles and liquids, excellent stability of diffusion with a predominance of Brownian movement of particles, and low pumping power in comparison to pure liquids to achieve intensification of equivalent heat transfer and reduce particle blockage in comparison to conventional pastes, thereby enhancing system compactness. Additionally, the Navier–Stockes model, which physically explains regular fluids, is present in micropolar fluids, commonly referred to as polar fluids. These fluids have a microstructure. They are fluids that experience asymmetrical stresses. Without being aware of the deformation of the fluid particles randomly suspended in a viscous liquid. Akbar et al.^1^ conducted the first study of nanofluid peristaltic flow by analyzing the temperature and nanoparticle equations using the (HPM). In a channel with suitable walls, Reddy and Reddy^2^ investigated the impact of the magnetic field and Joule heating on the peristaltic nanofluid. Ayub et al.^3^ were able to find a solution to the issue of peristaltic transport of MHD third-grade nanofluid in a curved channel while being affected by both thermal radiation and chemical reaction. Sucharitha et al.^4^ studied the mass and heat transfer properties of nanofluids peristaltic transport within a tapered channel. Instead of using the conventional linear radiation, Hayat et al.^5^ premeditated the magneto peristalsis of Jeffrey nanomaterial. Furthermore, Comprehensive Researches are available for this fertile field sees^6–12^.

Peristaltic transport is the term used to describe the fluid flow caused by waves travelling along a channel's sides. This definition is crucial when deciding whether to outlaw all pumping devices with pressure differences. Biomedical engineers have created numerous artificial devices such as blood pumps, dialysis machines, and other various applications because it has been observed that pumping vital fluids in many physiological systems such as blood flow in the blood vessels, pumping sperm into the ducts, transporting urine through the ureters, and swallowing food through the esophagus and through the peristaltic pumping principle. The force law model was taken into consideration when El-Dabe et al.^13^ identified the peristaltic motion of steady non-Newtonian nanofluid flow that obeyed through a non-Darcy porous medium. The effects of the magnetic field, buoyancy forces, thermophoresis, and Brownian motion on the peristaltic flow of the incompressible Jeffrey nanofluid were carefully examined by Reddy and Makinde^14^. Abd-Alla et al.^15^ elucidated the peristaltic flow of the Newtonian blood fluid model along an inclined asymmetric channel. The study of micropolar flow with allowance for thermal radiation through a resistive porous medium between channel walls was presented by Ahmad et al.^16^. The effects of the fractional Maxwell fluids on peristaltic flows within a circular cylinder tube were assessed by Bayones et al.^17^. To compare the combined peristalsis and electroosmosis-driven flow of silicon dioxide-water nanofluid and silver-water nanofluid, Akram et al.^18^ conducted a comparative experiment. By Ali and Hayat^19^, it was demonstrated that an incompressible micropolar fluid was moving peristaltically in an asymmetric conduit. The peristaltic transport of nanofluid in a conduit with compliant walls was being studied by Mustafa et al.^20^. El-Dabe and Shawky^21^ displayed how Dufour and Soret numbers affected on the peristaltic motion of a non‐Newtonian micropolar fluid.

Nowadays, there are numerous engineering, biological, and industrial uses for heat and mass transfer in peristaltic transport, including heat conduction caused by blood flow in tissues, biomass transfer, heat creation, and hypothermia to another. Numerous writers have investigated how heat and mass transport affect peristalsis. Following the researchers' foundational findings^22,23^, numerous works have been produced to describe how peristalsis behaves under various settings. The effects of electro-magneto-hydrodynamics, Hall currents, convective and slip boundary conditions, and peristaltic propulsion of nanofluids in porous symmetric microchannels were investigated by Ramesh et al.^24^. Additionally, numerous sources^24–30^ discuss some current study on this subject.

Numerous fields, including engineering, medicine, geophysics, and the oil industry, use flow via porous media. Peristaltic flow in porous media is a crucial research area because of recent advancements and needs in fluid mechanics, particularly in biomedical engineering and the sciences. The effects of heat absorption, chemical reaction, and wall characteristics on the peristaltic stream of a micropolar nanofluid via a permeable media were clarified by El-Dabe and Ramadan^31^. With the assumption that the wave is exceptionally long and has a low Reynolds number, Abd-Alla et al.^32^ provided a solution to the issue of the interaction between heat and mass transfer in the peristaltic flow of a second-grade fluid under the influence of a magnetic field through a tube. Additionally, numerical computation of Tripathi et al.^33^ was used to examine the double-diffusive convection in the flow of micropolar nanofluids. See Refs.^14,34–49^ for a discussion of a comparison analysis with earlier study findings.

The novelty of this study lies in showing how MHD micropolar nanofluid in an asymmetric channel is impacted by Brownian motion, buoyancy forces, thermophoresis, and heat and mass transfer. The system of partial differential equations governing this problem is first reduced to a handful of non-linear ordinary differential equations, which are then numerically solved utilizing the Rung–Kutta technique under the assumption of a long-wavelength and low Reynolds number approximation. Velocity profiles, temperature, concentration, microrotation, pressure gradient, the skin friction coefficient, Nusselt number, and Sherwood number, special emphasis is placed. The findings are analyzed and displayed graphically. Additionally, the drive stems from a desire to comprehend bio-magnetic fluid dynamics, a recent branch of fluid mechanics. These fluids have a wide range of uses in bioengineering and the medical fields. It is well known that this research resolves the issue of incompressible micropolar nanofluid and that, because of their varied uses and applications, nanofluids of tiny size gold particles drift in free have a significant impact on a variety of technological industries, including industrial production, scientific research, and various engineering sectors. The publicized findings are helpful for enhancing incandescent light bulbs' heating and cooling capabilities, the ability of the light-emitting filament, energy production, and a range of other heating devices. The current study analyses the effect of different parameters on peristaltic flow and we compare the results in form of graphs. Nanofluid consideration has been magnified due to their exceptional heat transfer characteristics and prospective applications in engineering and medical sciences after the pioneering work of Choi.

## Problem’s formulation

### Model description

**Figure 1:**
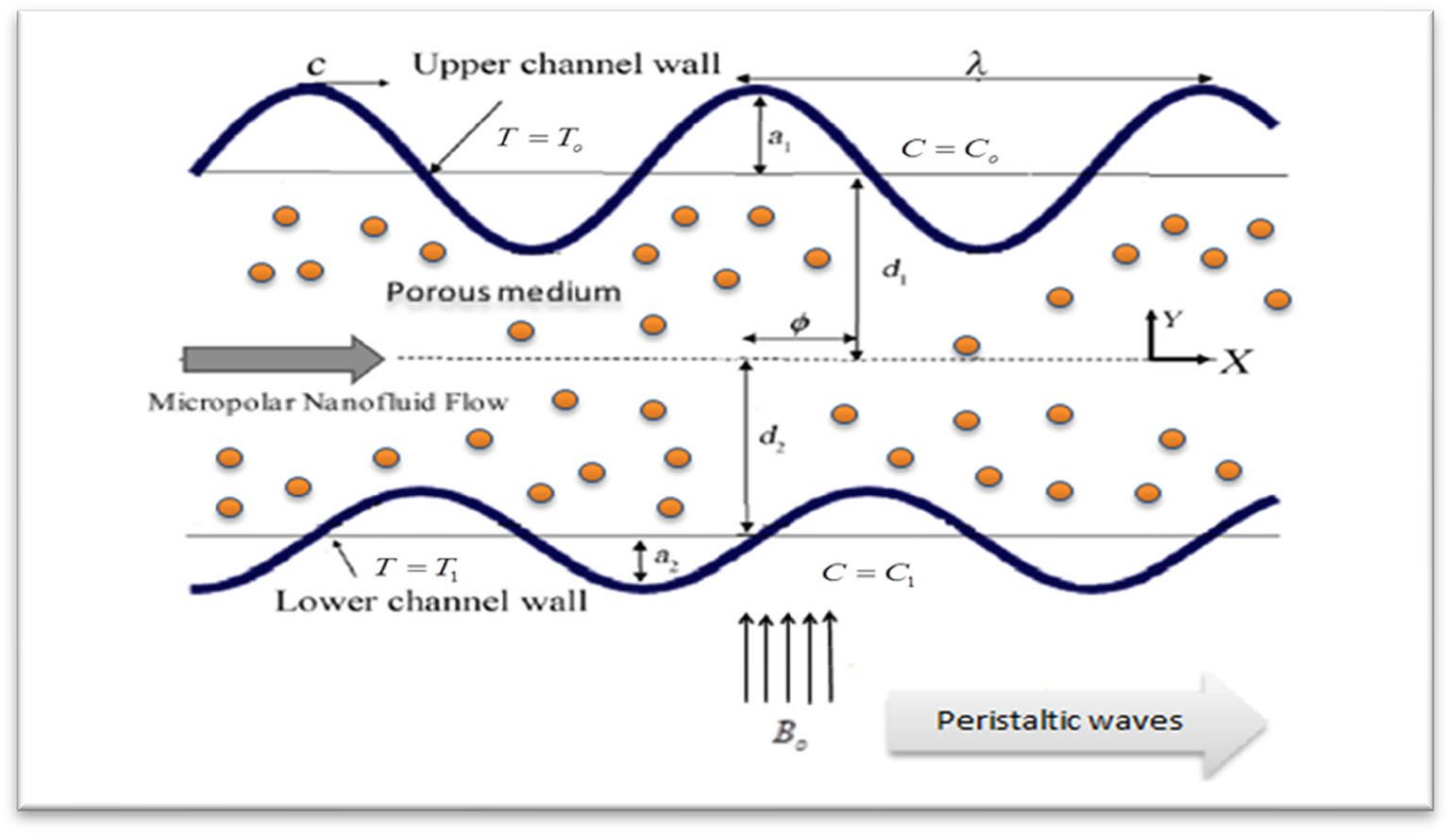
Geometrical illumination of the physical problem.

Contemplate the peristaltic flow of a width-asymmetric micropolar nanofluid that conducts electricity with the tiny size gold particles (GNPs) drift in space, A sinusoidal wave that is propagating down the channel's walls at constant speed $c$ is what causes the peristaltic motion ($\tilde{\chi }_{2}$ is the lower wall and $\tilde{\chi }_{1}$ is the upper wall. According to Fig. 1.

### Governing equations

The asymmetric channel's upper and lower wall margins are $Y = \tilde{\chi }_{1} ,$ and $Y = \tilde{\chi }_{2}$ respectively. The present mathematical analysis is considered under presumptions of long-wavelength and Low Reynolds numbers. The two wall surfaces can be described mathematically using the equations below^19^:

1 $$\tilde{\chi }_{1} = d_{1} + a_{1} \cos \left[ {\frac{2\pi }{\lambda }\left( {\tilde{X} - c\tilde{t}} \right)} \right],$$

2 $$\tilde{\chi }_{2} = - d_{2} - b_{1} \cos \left[ {\frac{2\pi }{\lambda }\left( {\tilde{X} - c\tilde{t}} \right) + \varphi } \right].$$

In the above terminology, $\tilde{X}$ and $\tilde{Y}$ are the direction propagation and its perpendicular to it in this case. Depending on which is used, the phase difference $\varphi$ changes. Out of phase $\left( {\varphi = 0} \right)$ related to symmetric channels and in phase $\left( {\varphi = \pi } \right)$ relate to asymmetric channels. The flow receives an even magnetic field application. To make the induced magnetic field insignificant in comparison to the applied magnetic field, the magnetic Reynolds number is taken to be small and the electric field to be zero. Also, $a_{1} ,b_{1} ,d_{1} ,d_{2}$ meet the following criterion^15^:

3 $$a_{1}^{2} + b_{1}^{2} + 2a_{1} b_{1} \cos \varphi \le \left( {d_{1} + d_{2} } \right)^{2} .$$

The formula for the incompressible material's Cauchy stress tensor ${\rm T}$ is^14^:

4 $${\rm T} = - PI + S,\,\,S = \frac{\mu }{{1 + \lambda_{1} }}\left( {\mathop \gamma \limits^{.} + \lambda_{2} \frac{{d\mathop \gamma \limits^{.} }}{dt}} \right),$$

where, ${\rm T}$ is stress tensor, $S$ is Cauchy extra stress tensor, and $I$ is the identity tensor.

The regulating equations of the flow of an incompressible micropolar nanofluid in a fixed frame are^14,28^:

5 $$\frac{{\partial \tilde{\xi }}}{{\partial \tilde{X}}} + \frac{{\partial \tilde{\zeta }}}{{\partial \tilde{Y}}} = 0,$$

6 $$\begin{aligned} & \rho_{f} \left[ {\frac{{\partial \tilde{\xi }}}{{\partial \tilde{t}}} + \tilde{\xi }\frac{{\partial \tilde{\xi }}}{{\partial \tilde{X}}} + \tilde{\zeta }\frac{{\partial \tilde{\xi }}}{{\partial \tilde{Y}}}} \right] = - \frac{{\partial \overline{P} }}{{\partial \tilde{X}}} + \frac{\partial }{{\partial \tilde{X}}}\left( {\tilde{S}_{{\tilde{X}\tilde{X}}} } \right) + \frac{\partial }{{\partial \tilde{Y}}}\left( {\tilde{S}_{{\tilde{X}\tilde{Y}}} } \right) + (1 - C_{o} )\rho_{f} g\varsigma_{t} \left( {\tilde{T} - T_{o} } \right) \\ & \quad + \left( {\frac{{\rho_{p} - \rho_{f} }}{{\rho_{f} }}} \right)g\varsigma_{c} \left( {\tilde{C} - C_{o} } \right) - \sigma B_{o}^{2} \tilde{\xi } - \frac{\mu }{{k_{1} }}\tilde{\xi } + k\frac{{\partial \tilde{W}}}{{\partial \tilde{Y}}}, \\ \end{aligned}$$

7 $$\rho_{f} \left[ {\frac{{\partial \tilde{\zeta }}}{{\partial \tilde{t}}} + \tilde{\xi }\frac{{\partial \tilde{\zeta }}}{{\partial \tilde{X}}} + \tilde{\zeta }\frac{{\partial \tilde{\zeta }}}{{\partial \tilde{Y}}}} \right] = - \frac{{\partial \overline{P} }}{{\partial \tilde{Y}}} + \frac{\partial }{{\partial \tilde{X}}}\left( {\tilde{S}_{{\tilde{X}\tilde{Y}}} } \right) + \frac{\partial }{{\partial \tilde{Y}}}\left( {\tilde{S}_{{\tilde{Y}\tilde{Y}}} } \right) - \frac{\mu }{{k_{1} }}\tilde{\zeta } + k\frac{{\partial \tilde{W}}}{{\partial \tilde{X}}},$$

8 $$\begin{aligned} & \left[ {\frac{{\partial \tilde{T}}}{{\partial \tilde{t}}} + \tilde{\xi }\frac{{\partial \tilde{T}}}{{\partial \tilde{X}}} + \tilde{\zeta }\frac{{\partial \tilde{T}}}{{\partial \tilde{Y}}}} \right] = \alpha \left( {\frac{{\partial^{2} \tilde{T}}}{{\partial \tilde{X}^{2} }} + \frac{{\partial^{2} \tilde{T}}}{{\partial \tilde{Y}^{2} }}} \right) + \frac{1}{{\rho_{f} c_{f} }}\left[ {\tilde{S}_{{\tilde{X}\tilde{X}}} \frac{{\partial \tilde{\xi }}}{{\partial \tilde{X}}} + \tilde{S}_{{\tilde{X}\tilde{Y}}} \left( {\frac{{\partial \tilde{\xi }}}{{\partial \tilde{Y}}} + \frac{{\partial \tilde{\zeta }}}{{\partial \tilde{X}}}} \right) + \tilde{S}_{{\tilde{Y}\tilde{Y}}} \frac{{\partial \tilde{\zeta }}}{{\partial \tilde{X}}}} \right] \\ & \quad + \tau \left[ {D_{B} \left( {\frac{{\partial \tilde{C}}}{{\partial \tilde{X}}}\frac{{\partial \tilde{T}}}{{\partial \tilde{X}}} + \frac{{\partial \tilde{C}}}{{\partial \tilde{Y}}}\frac{{\partial \tilde{T}}}{{\partial \tilde{Y}}}} \right) + \frac{{D_{T} }}{{T_{m} }}\left( {\left( {\frac{{\partial \tilde{T}}}{{\partial \tilde{X}}}} \right)^{2} + \left( {\frac{{\partial \tilde{T}}}{{\partial \tilde{Y}}}} \right)^{2} } \right)} \right] + \frac{{\sigma B_{o}^{2} \tilde{\xi }^{2} }}{{\rho_{f} c_{f} }}, \\ \end{aligned}$$

9 $$\left[ {\frac{{\partial \tilde{C}}}{{\partial \tilde{t}}} + \tilde{\xi }\frac{{\partial \tilde{C}}}{{\partial \tilde{X}}} + \tilde{\zeta }\frac{{\partial \tilde{C}}}{{\partial \tilde{Y}}}} \right] = D_{B} \left( {\frac{{\partial^{2} \tilde{C}}}{{\partial \tilde{X}^{2} }} + \frac{{\partial^{2} \tilde{C}}}{{\partial \tilde{Y}^{2} }}} \right) + \frac{{D_{T} }}{{T_{m} }}\left( {\frac{{\partial^{2} \tilde{T}}}{{\partial \tilde{X}^{2} }} + \frac{{\partial^{2} \tilde{T}}}{{\partial \tilde{Y}^{2} }}} \right),$$

10 $$\rho_{f} \overline{J} \left[ {\frac{{\partial \tilde{W}}}{{\partial \overline{t} }} + \tilde{\xi }\frac{{\partial \tilde{W}}}{{\partial \tilde{X}}} + \tilde{\zeta }\frac{{\partial \tilde{W}}}{{\partial \tilde{Y}}}} \right] = - 2k\tilde{W} + \gamma_{1} \left( {\frac{{\partial^{2} \tilde{W}}}{{\partial \tilde{X}^{2} }} + \frac{{\partial^{2} \tilde{W}}}{{\partial \tilde{Y}^{2} }}} \right) + k\left( {\frac{{\partial \tilde{\zeta }}}{{\partial \tilde{X}}} - \frac{{\partial \tilde{\xi }}}{{\partial \tilde{Y}}}} \right),$$

where $\tilde{S}_{{\tilde{X}\tilde{X},}} \tilde{S}_{{\tilde{X}\tilde{Y},}} \,\tilde{S}_{{\tilde{Y}\tilde{Y}}}$ are the extra stress tensor components, and $\tau = \tfrac{{(\rho c)_{p} }}{{(\rho c)_{f} }}$ is the ratio of the nanoparticle material's effective heat capacity to the fluid's heat capacity.

In the wave frame $(\tilde{x},\tilde{y})$, the flow is supposed to be steady and travel away from the fixed frame $(\tilde{X},\tilde{Y})$ at a constant rate. The following are the conversions between wave frame and laboratory frame^15^:

11 $$\tilde{x} = \tilde{X} + c\tilde{t},\tilde{y} = \tilde{Y},\tilde{u} = \tilde{\xi } - c,\tilde{v} = \tilde{\zeta },\tilde{p}(\tilde{x},\tilde{y}) = \tilde{P}(\tilde{X},\tilde{Y},\tilde{t}),\tilde{w}\left( {\tilde{x},\tilde{y}} \right) = \tilde{W}(\tilde{X},\tilde{Y},\tilde{t}),T = \tilde{T}.$$

These non-dimensional parameters and variables should be imposed as follows^14,28^:

12 $$\begin{aligned} & x = \frac{{\tilde{x}}}{\lambda },y = \frac{{\tilde{y}}}{{d_{1} }},u = \frac{{\tilde{u}}}{c},v = \frac{{\tilde{v}}}{c},t = \frac{{c\tilde{t}}}{\lambda },l_{1} = \frac{{\tilde{\chi }_{1} }}{{d_{1} }}, \\ & l_{2} = \frac{{\tilde{\chi }_{2} }}{{d_{2} }},a = \frac{{a_{1} }}{{d_{1} }},b = \frac{{b_{1} }}{{d_{1} }},d = \frac{{d_{2} }}{{d_{1} }},\delta^{\prime} = \frac{{d_{1} }}{\lambda },p = \frac{{d_{1}^{2} \tilde{p}}}{c\lambda \mu }, \\ & M = \sqrt {\frac{\sigma }{\mu }} B_{0} d_{1} ,\overline{{\lambda_{2} }} = \frac{{\lambda_{2} c}}{{d_{1} }},\Pr = \frac{\upsilon }{\alpha },{\text{Re}} = \frac{{cd_{1} }}{\upsilon },S = \frac{{\tilde{S}d_{1} }}{\mu c}, \\ & Gm = \frac{{\left( {\rho_{c} - \rho_{f} } \right)g\varsigma_{c} \left( {C_{1} - C_{o} } \right)d_{1}^{2} }}{\mu c},Nt = \frac{{\tau D_{T} (T_{1} - T_{o} )}}{{\upsilon T_{o} }},Nb = \frac{{\tau D_{B} (C_{1} - C_{o} )}}{\upsilon }, \\ & Gr = \frac{{\left( {1 - C_{o} } \right)\rho_{f} g\varsigma_{t} \left( {T_{1} - T_{o} } \right)d_{1}^{2} }}{\mu c},Ec = \frac{{c^{2} }}{{c_{f} \left( {T_{1} - T_{0} } \right)}},\eta = \frac{{d_{1}^{2} }}{{k_{1} }} = \frac{1}{Da}, \\ & Br = \Pr \times Ec,\theta = \frac{{\tilde{T} - T_{0} }}{{T_{1} - T_{0} }},\Omega = \frac{{\tilde{C} - C_{0} }}{{C_{1} - C_{0} }},w = \frac{{\tilde{w}d_{1} }}{c},J = \frac{{\overline{J} }}{{d_{1}^{2} }}. \\ \end{aligned}$$

## Problem’s solution

The following equations can be made simpler by using non-dimensional variables:

13 $$\begin{aligned} & {\text{Re}} \delta^{\prime}\left[ {\left( {u + 1} \right)\frac{\partial u}{{\partial x}} + \frac{v}{{\delta^{\prime}}}\frac{\partial u}{{\partial y}}} \right] = - \frac{dp}{{dx}} + \delta^{\prime}\frac{\partial }{\partial x}\left( {S_{xx} } \right) + \frac{\partial }{\partial y}\left( {S_{xy} } \right) - \left( {M^{2} + \frac{1}{Da}} \right) \times \left( {u + 1} \right) \\ & \,\quad + Gr\theta + Gm\Omega + \frac{k}{\mu }\frac{\partial w}{{\partial y}}, \\ \end{aligned}$$

14 $${\text{Re}} \delta^{{\prime}{3}} \left[ {\left( {u + 1} \right)\frac{\partial v}{{\partial x}} + \frac{v}{{\delta^{\prime}}}\frac{\partial v}{{\partial y}}} \right] = - \frac{\partial p}{{\partial y}} + \delta^{{\prime}{2}} \frac{\partial }{\partial x}\left( {S_{xy} } \right) + \delta^{\prime}\frac{\partial }{\partial y}\left( {S_{yy} } \right) - \frac{{\delta^{\prime}}}{Da}v + \delta^{{\prime}{2}} \frac{k}{\mu }\frac{\partial w}{{\partial x}},$$

15 $$\begin{aligned} & {\text{Re}} \delta^{\prime}\left[ {\left( {u + 1} \right)\frac{\partial \theta }{{\partial x}} + \frac{v}{{\delta^{\prime}}}\frac{\partial \theta }{{\partial y}}} \right] = Ec\left[ {\delta^{\prime}\,S_{xx} \frac{\partial u}{{\partial x}} + S_{xx} \left( {\frac{\partial u}{{\partial y}} + \delta^{{\prime}{2}} \frac{\partial v}{{\partial x}}} \right) + \,S_{yy} \frac{\partial v}{{\partial y}} + M^{2} \left( {u + 1} \right)^{2} } \right] \\ & \quad + \frac{1}{\Pr }\left[ {\delta^{{\prime}{2}} \frac{{\partial^{2} \theta }}{{\partial x^{2} }} + \frac{{\partial^{2} \theta }}{{\partial y^{2} }}} \right] + Nb\left[ {\frac{\partial \theta }{{\partial y}}\frac{\partial \Omega }{{\partial y}} + \delta^{{\prime}{2}} \frac{\partial \theta }{{\partial x}}\frac{\partial \Omega }{{\partial x}}} \right] + Nt\left[ {\left( {\frac{\partial \theta }{{\partial y}}} \right)^{2} + \delta^{{\prime}{2}} \left( {\frac{\partial \theta }{{\partial x}}} \right)^{2} } \right], \\ \end{aligned}$$

16 $${\text{Re}} \delta^{\prime}\left[ {\left( {u + 1} \right)\frac{\partial \Omega }{{\partial x}} + \frac{v}{{\delta^{\prime}}}\frac{\partial \Omega }{{\partial y}}} \right] = \left( {\frac{{\partial^{2} \Omega }}{{\partial y^{2} }} + \delta^{{\prime}{2}} \frac{{\partial^{2} \Omega }}{{\partial x^{2} }}} \right) + \frac{Nt}{{Nb}}\left( {\frac{{\partial^{2} \theta }}{{\partial y^{2} }} + \delta^{{\prime}{2}} \frac{{\partial^{2} \theta }}{{\partial x^{2} }}} \right),$$

17 $$\rho_{f} Jd^{\prime}_{1} c\delta^{\prime}\left[ {\left( {u + 1} \right)\frac{\partial w}{{\partial x}} + \frac{v}{{\delta^{\prime}}}\frac{\partial w}{{\partial y}}} \right] = - 2kw + \frac{{\gamma_{1} }}{{d_{1}^{2} }}\left( {\delta^{{\prime}{2}} \frac{{\partial^{2} w}}{{\partial x^{2} }} + \frac{{\partial^{2} w}}{{\partial y^{2} }}} \right) + k\left( {\delta^{\prime}\frac{\partial v}{{\partial x}} - \frac{\partial u}{{\partial y}}} \right).$$

The definition of stream function $\psi^{\prime}$ and velocities are inserted here now

18 $$u = \frac{{\partial \psi^{\prime}}}{\partial y},\,\,\,\,\,\,\,\,v = - \delta^{\prime}\frac{{\partial \psi^{\prime}}}{\partial x}.$$

The stresses components in this system are represented by the following equations^14^:

19 $$S_{xx} = \frac{{2\delta^{\prime}}}{{\left( {1 + \lambda_{1} } \right)}}\left[ {1 + \frac{{\lambda_{2} c\delta^{\prime}}}{{d^{\prime}_{1} }}\left( {\psi^{\prime}_{y} \frac{\partial }{\partial x} - \psi^{\prime}_{x} \frac{\partial }{\partial y}} \right)} \right]\psi^{\prime}_{xy} ,$$

20 $$S_{xy} = \frac{1}{{\left( {1 + \lambda_{1} } \right)}}\left[ {1 + \frac{{\lambda_{2} c\delta^{\prime}}}{{d^{\prime}_{1} }}\left( {\psi^{\prime}_{y} \frac{\partial }{\partial x} - \psi^{\prime}_{x} \frac{\partial }{\partial y}} \right)} \right]\left( {\psi^{\prime}_{yy} - \delta^{{\prime}{2}} \psi^{\prime}_{xx} } \right),$$

21 $$S_{yy} = \frac{{2\delta^{\prime}}}{{\left( {1 + \lambda_{1} } \right)}}\left[ {1 + \frac{{\lambda_{2} c\delta^{\prime}}}{{d_{1} }}\left( {\psi^{\prime}_{y} \frac{\partial }{\partial x} - \psi^{\prime}_{x} \frac{\partial }{\partial y}} \right)} \right]\psi^{\prime}_{xy} .$$

Non-dimensional regulating flow Eqs. (13)–(17) are solved using long-wavelength and low Reynolds number approaches, as follows:

22 $$- \frac{dp}{{dx}} + \left( {\frac{1}{{1 + \lambda_{1} }}} \right)\frac{{\partial^{3} \psi^{\prime}}}{{\partial y^{3} }} + Gr\theta + Gm\Omega - \left( {M^{2} + \frac{1}{Da}} \right)\left[ {\frac{{\partial \psi^{\prime}}}{\partial y} + 1} \right] + \frac{k}{\mu }\frac{\partial w}{{\partial y}} = 0,$$

23 $$- \frac{\partial p}{{\partial y}} = 0,$$

24 $$\frac{{\partial^{2} \theta }}{{\partial y^{2} }} + Br\left[ {\left( {\frac{1}{{1 + \lambda_{1} }}} \right)\left( {\frac{{\partial^{2} \psi^{\prime}}}{{\partial y^{2} }}} \right)^{2} + M^{2} \left( {\frac{{\partial \psi^{\prime}}}{\partial y} + 1} \right)^{2} } \right] + \Pr Nb\frac{\partial \theta }{{\partial y}}\frac{\partial \Omega }{{\partial y}} + \Pr Nt\left( {\frac{\partial \theta }{{\partial y}}} \right)^{2} = 0,$$

25 $$\frac{{\partial^{2} \Omega }}{{\partial y^{2} }} + \frac{Nt}{{Nb}}\frac{{\partial^{2} \theta }}{{\partial y^{2} }} = 0,$$

26 $$- 2w - \frac{{\partial^{2} \psi }}{{\partial y^{2} }} + \frac{{\gamma_{1} }}{{d_{1}^{\prime 2} k}}\frac{{\partial^{2} w}}{{\partial y^{2} }} = 0.$$

Equations (22) and (23) can be reconciled by removing pressure using cross differentiation as:

27 $$\left( {\frac{1}{{1 + \lambda_{1} }}} \right)\frac{{\partial^{4} \psi^{\prime}}}{{\partial y^{4} }} - \left( {M^{2} + \frac{1}{Da}} \right)\frac{{\partial^{2} \psi^{\prime}}}{{\partial y^{2} }} + \frac{k}{\mu }\frac{{\partial^{2} w}}{{\partial y^{2} }} + Gr\frac{\partial \theta }{{\partial y}} + Gm\frac{\partial \Omega }{{\partial y}} = 0.$$

The following are the definitions of the applicable regulated boundary conditions:

28 $$\psi^{\prime} = \frac{q}{2},\frac{{\partial \psi^{\prime}}}{\partial y} = - 1,\,\,\theta = 0,\,\,\Omega = 0,\,\,w = 0\,\,\,\,\,\,at\,\,\,\,y = l_{1} = 1 + a\cos \left( {2\pi x} \right),$$

29 $$\psi^{\prime} = - \frac{q}{2},\frac{{\partial \psi^{\prime}}}{\partial y} = - 1,\,\,\theta = 1,\,\,\Omega = 1,\,\,w = 0\,\,\,\,\,\,at\,\,\,\,y = r_{2} = - d - b\cos \left( {2\pi x + \varphi } \right).$$

Wave and fixed frame flow rates can be linked using the following equations:

30 $$Q = q + 1 + d.$$

The shear stress, heat flux, and volume flow of nanoparticles in the channel walls are computed as follows^14^:

31 $$C_{f} = \left. {\left( {\frac{1}{{1 + \lambda_{1} }}} \right)\frac{\partial u}{{\partial y}}} \right|_{{y = l_{1} ,l_{2} }} ,\,\,\,\,Nu = - \left. {\frac{\partial \theta }{{\partial y}}} \right|_{{y = l_{1} ,l_{2} }} ,\,\,\,\,\,\,Sh = - \left. {\frac{\partial \Omega }{{\partial y}}} \right|_{{y = l_{1} ,l_{2} }} .$$

## Solution methodology

The present part points out the used numerical method for solving (24)–(27) related to the boundary conditions (28) and (29). Which are very nonlinear and their solutions are simply not feasible in their closed form. This dilemma can be dissolved numerically by the Runge–Kutta–Fehlberg (RK4) method with various parameter values formally known and described in the book by Zheng and Zhang^33^. The stride size is taken small and accuracy reaches the 4th tenth point as the convergence precept. We assumed a suitable limited value for the far domain boundary condition in (28) and (29).

These resulting governing Eqs. (24)–(27) of the micropolar nanofluid model are coupled and highly nonlinear. Getting the exact solution is impossible. Therefore, the numerical solution has been obtained. The numerical calculations have been obtained using the MATLAB software. Moreover, an excellent agreement is found can be seen in Tables.

## Numerical simulation procedure

The obtaining nonlinear higher order of ODEs is minimized to 1^st^ order differential structures by presenting the new variables as:

32 $$\left. \begin{gathered} \Psi^{\prime} = f_{1} ,\,\,\,\,\,D\Psi^{\prime} = f_{2} ,\,\,\,\,\,DD\Psi^{\prime} = f_{3} ,\,\,\,\,\,DDD\psi^{\prime} = f_{4} , \hfill \\ \theta = f_{5} ,\,\,\,\,D\theta = f_{6} ,\,\,\,\,\Omega = f_{7} ,\,\,\,\,D\Omega = f_{8} ,\,\,\,\,w = f_{9} ,\,\,\,Dw = f_{10} \hfill \\ \end{gathered} \right\}$$

The coupled higher order differential equations and the boundary conditions may be transformed into ten equivalent first order differential equations:

33 $$\left. \begin{aligned} & F_{1} = Y_{2} ,\,\,\,\,\,F_{2} = Y_{3} ,\,\,\,\,\,\,F_{3} = Y_{4} , \hfill \\ & F_{4} = \left( { - Gr\,Y_{6} - Gm\,Y_{8} - \left( {\left( \frac{1}{Da} \right) + M^{2} } \right) \times Y_{3} - \left( {\left( {\frac{k}{\mu }} \right)\, \times Y_{10} } \right)} \right) \times \left( {1 + \lambda_{1} } \right),\,\,\,\,\,F_{5} = Y_{6} , \hfill \\ & F_{6} = - Br \times \left( {\left( {\frac{1}{{\left( {1 + \lambda } \right)}}} \right) \times Y_{3}^{2} + M^{2} \times \left( {Y_{2} + 1} \right)^{2} } \right) - \Pr \,Nb\, \times Y_{6} \times Y_{8} - \Pr Nt \times Y_{6}^{2} ,\,\,\,F_{7} = Y_{8} , \hfill \\ & F_{8} = \frac{{ - Nt\, \times F_{6} }}{Nb},\,\,\,\,\,F_{9} = Y_{10} ,\,\,\,\,\,F_{10} = \frac{{\left( {2\,Y_{9} + Y_{3} } \right)\,d_{1}^{2} \times k}}{{\gamma_{1} }}. \hfill \\ \end{aligned} \right\}$$

## Results and discussion

**Figure 2:**
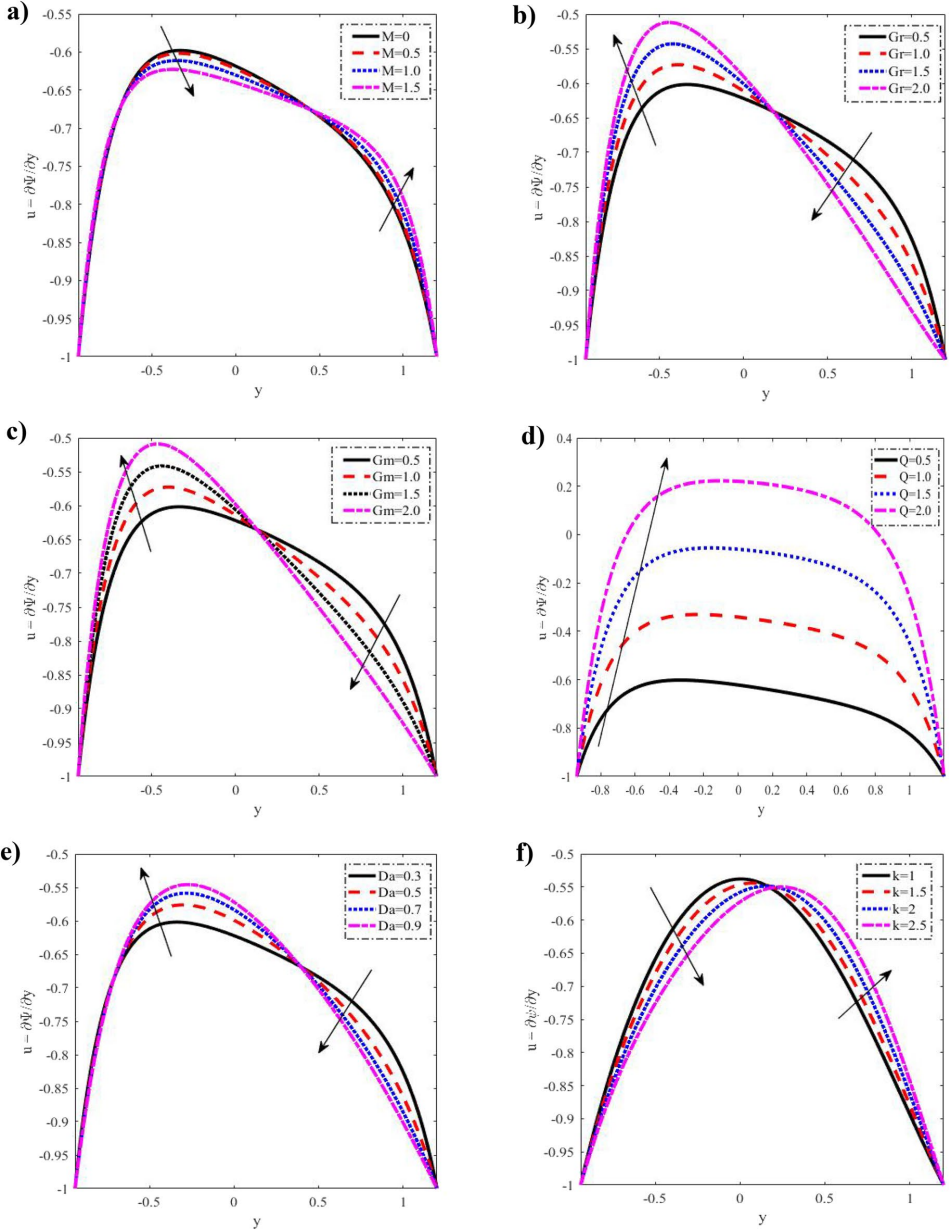
Discrepancies of the velocity *u* against the y-axis. (**a**) For different values of $M.$ (**b**) For different values of $Gr.$ (**c**) For different values of $Gm.$ (**d**) For different values of $Q.$ (**e**) For different values of $Da.$ (**f**) For different values of $k.$ When $x = 0.1,a = 0.3,b = 0.5,d = 1,\phi = \frac{\pi }{3},k = 1,Gr = 0.5,\lambda_{1} = 0.5,Br = 0.1.\Pr = 1,Nt = 0.2,Nb = 0.2,q = - 1.5,\mu = 0.2,Gm = 0.5,d_{1} = 0.3,\gamma_{1} = 0.5,Da = 0.3,Ec = 0.1,M = 0.5.$

**Figure 3:**
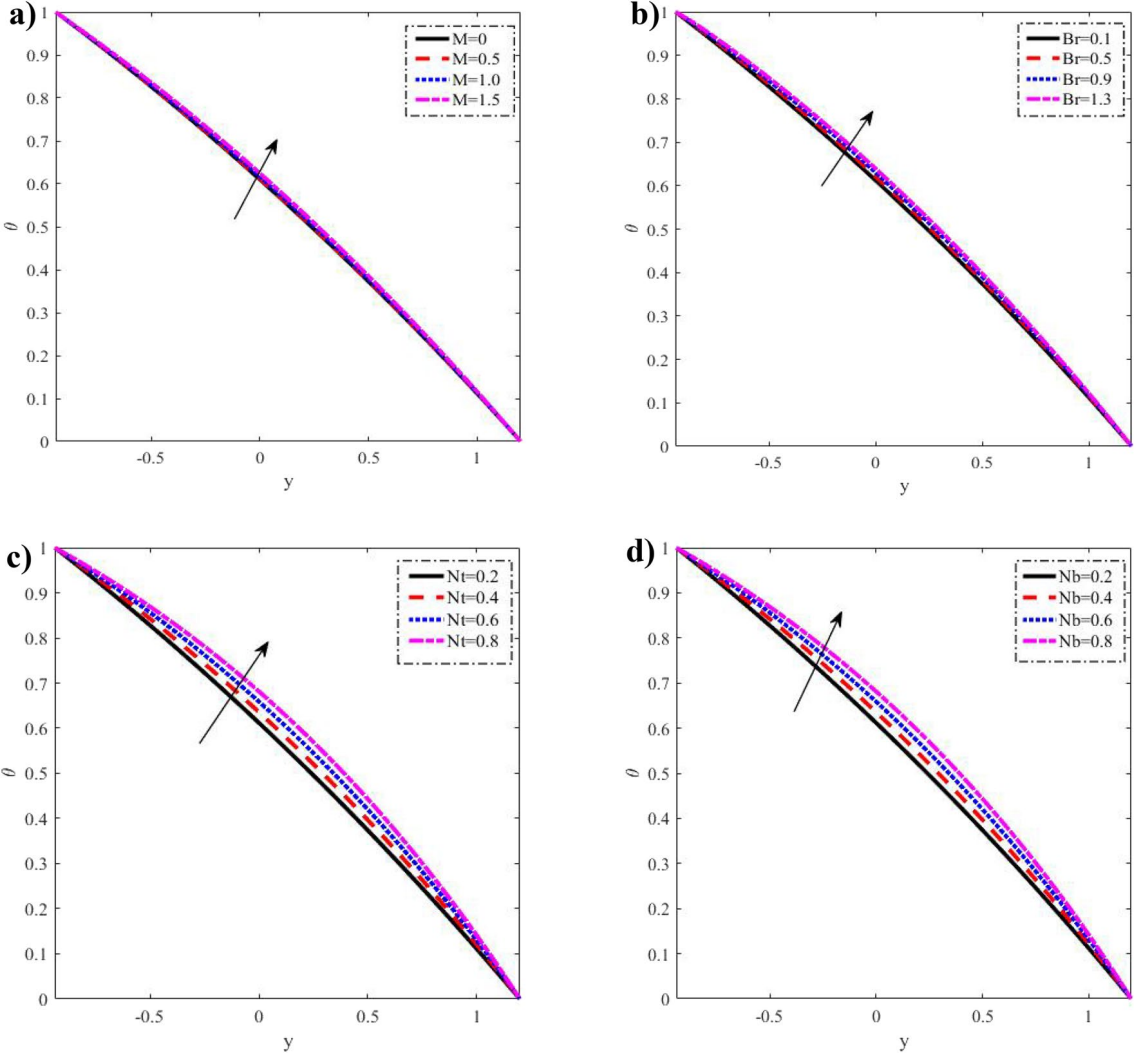
Discrepancies of the temperature _θ_ against the y-axis. (**a**) For different values of $M.$ (**b**) For different values of $Br.$ (**c**) For different values of $Nt.$ (**d**) For different values of $Nb.$

**Figure 4:**
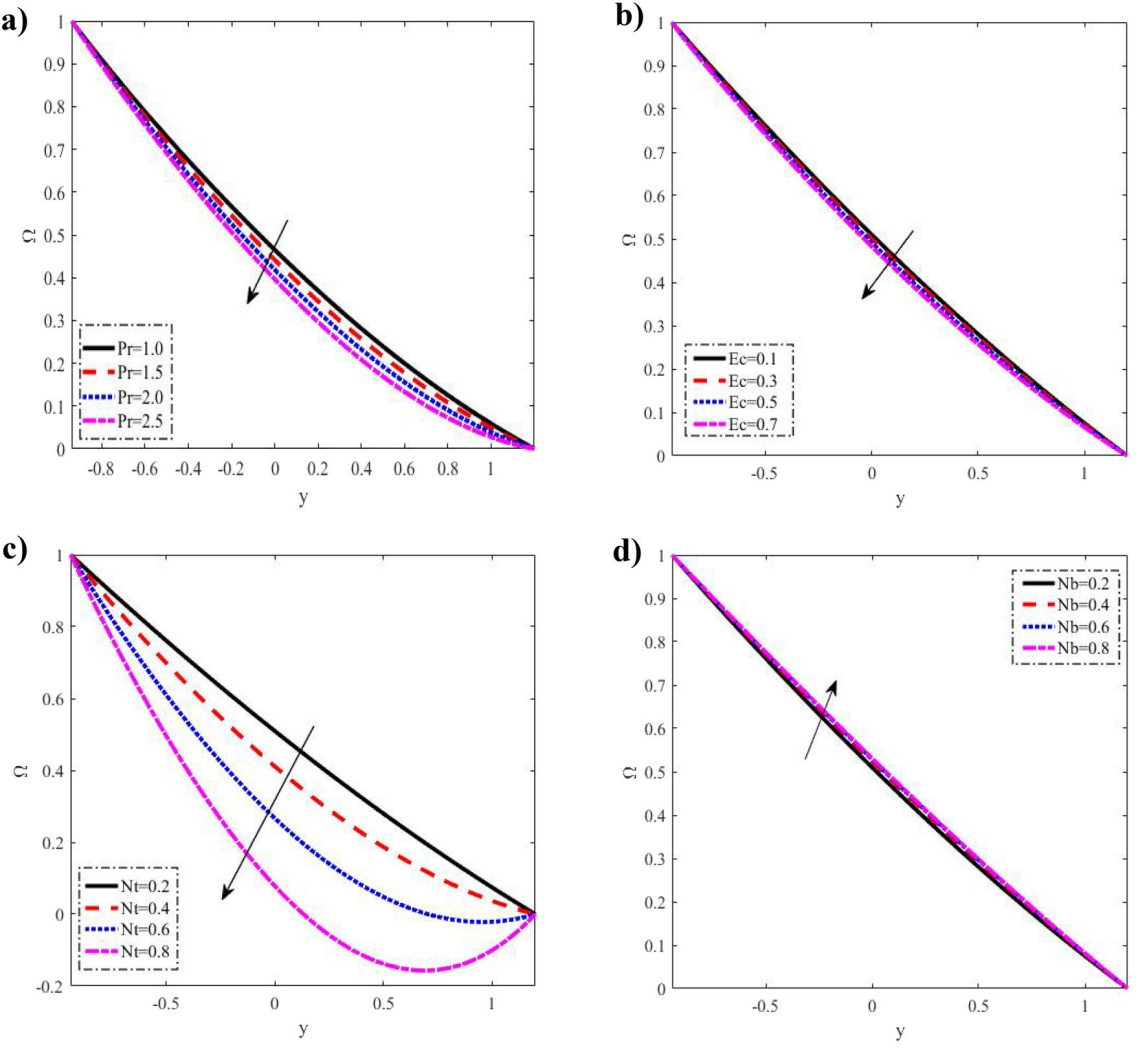
Discrepancies of the concentration $\Omega$ against the y-axis. (**a**) For different values of $\Pr .$ (**b**) For different values of $Ec.$ (**c**) For different values of $Nt.$ (**d**) For different values of $Nb.$

**Figure 5:**
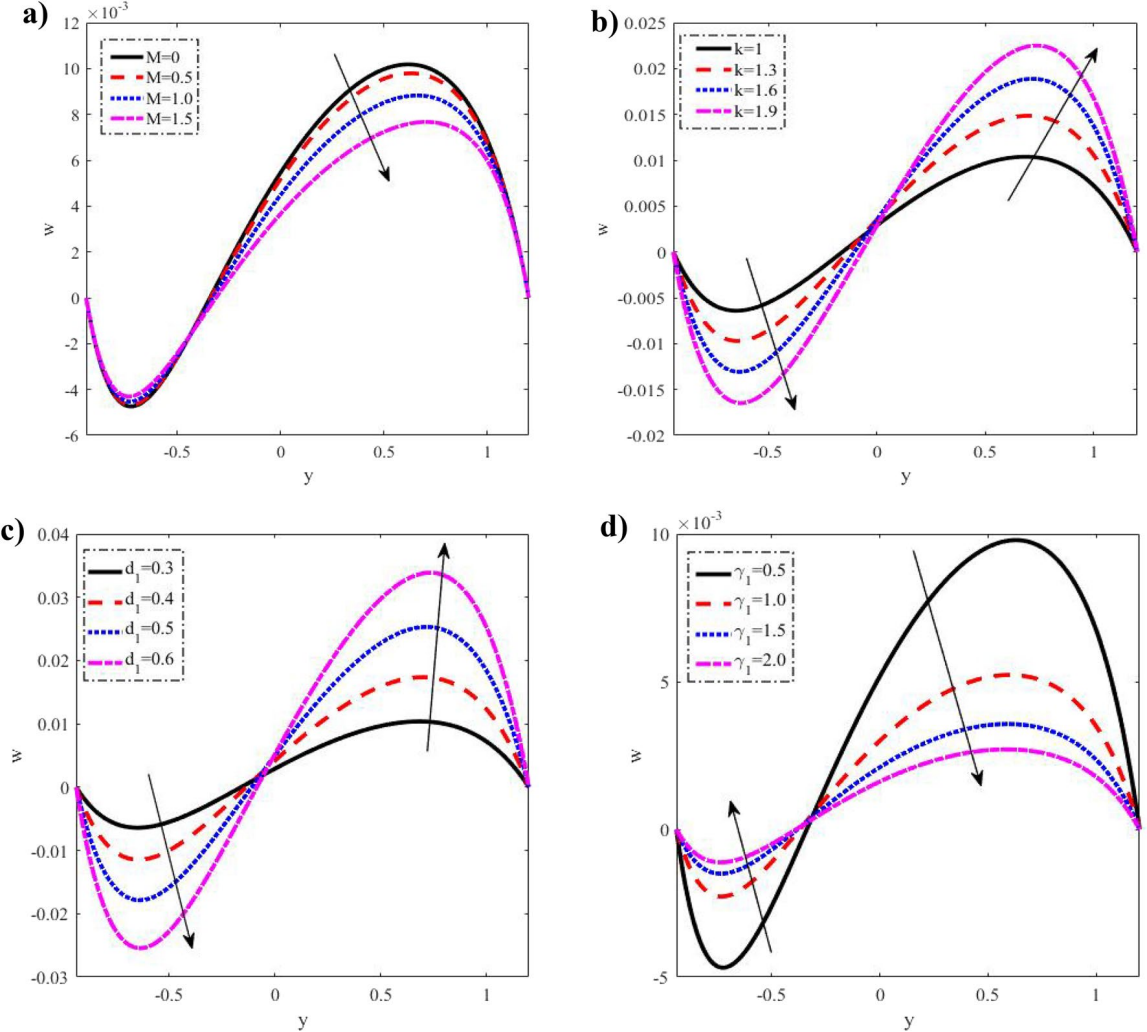
Discrepancies of the microrotation velocity $w$ against the y-axis. (**a**) For different values of $M.$ (**b**) For different values of $k.$ (**c**) For different values of $d_{1} .$ (**d**) For different values of $\gamma_{1} .$

**Figure 6:**
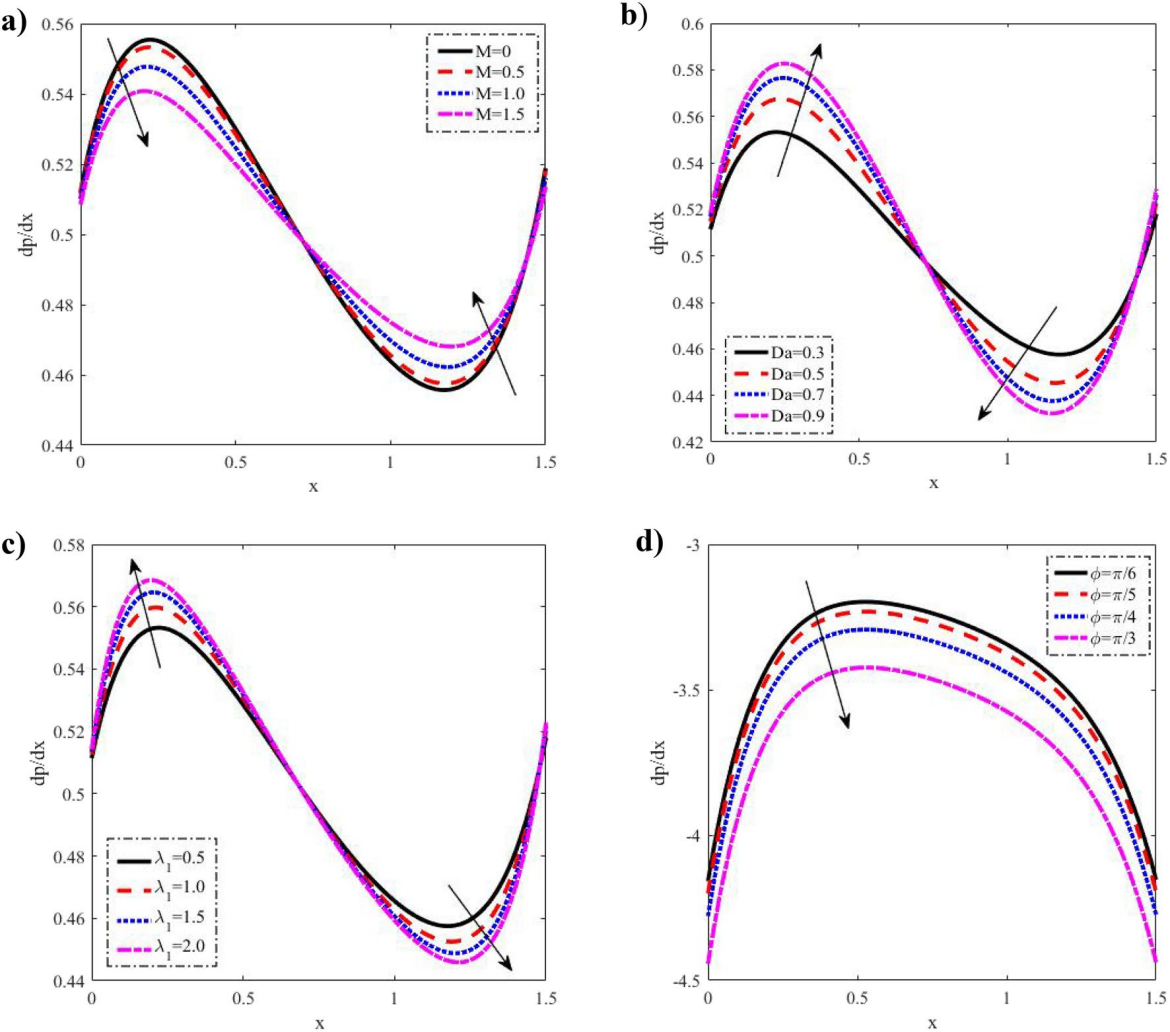
Discrepancies of the pressure gradient $\frac{dp}{{dx}}$ against the *x*-axis. (**a**) For different values of $M.$ (**b**) For different values of $Da.$ (**c**) For different values of $\lambda_{1} .$ (**d**) For different values of $\varphi .$

**Figure 7:**
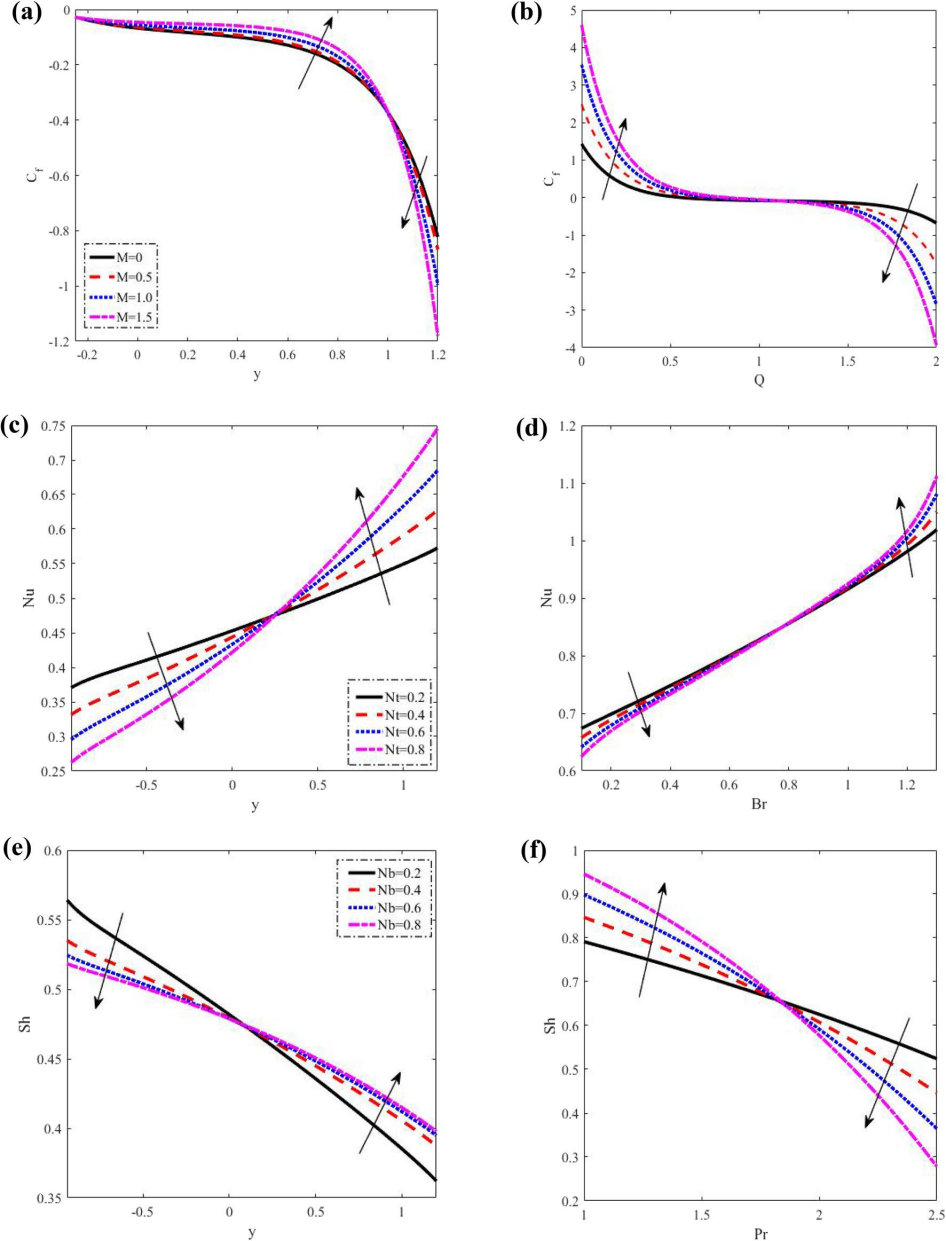
Discrepancies of the shear stress *C*_*f*_ the heat flux *Nu* and the nanoparticle volume flux *Sh*. (**a**) Discrepancies of *C*_*f*_ against *M*. (**b**) Discrepancies of *C*_*f*_ against *Q*. (**c**) Discrepancies of *Nu* against *Nt*. (**d**) Discrepancies of *Nu* against *Br*. (**e**) Discrepancies of *Sh* against *Nb*. (**f**) Discrepancies of *Sh* against Pr.

**Figure 8:**
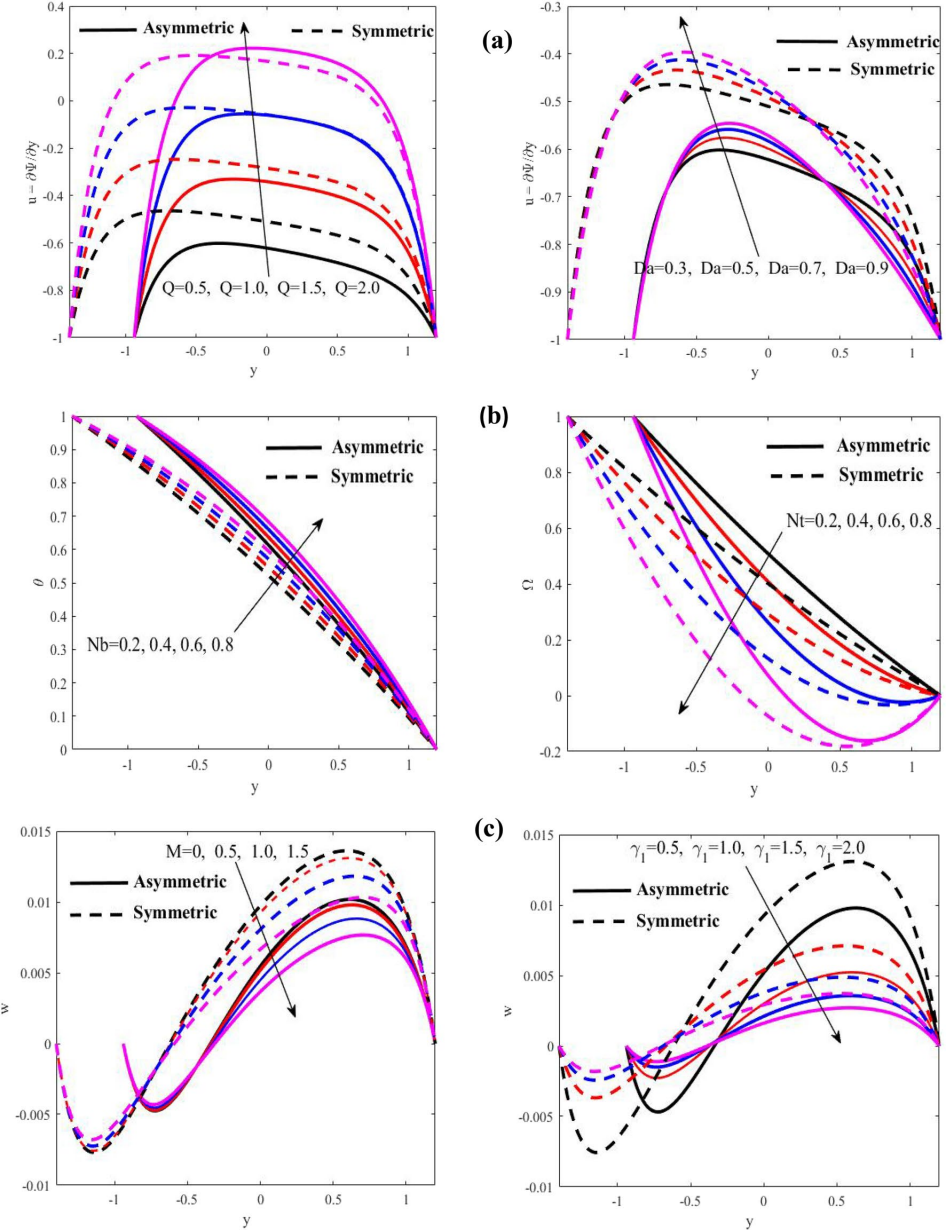
Discrepancies of the velocity *u*, the temperature *θ*, and the microrotation velocity *w* in two different cases. (**a**) Discrepancies of *u* against *Q* and *Da*. (**b**) Discrepancies of $\theta$ and $\Theta$ against $Nb$ and $Nt,$ respectively. (**c**) Discrepancies of $w$ against $M$ and $\gamma_{1} .$

**Figure 9:**
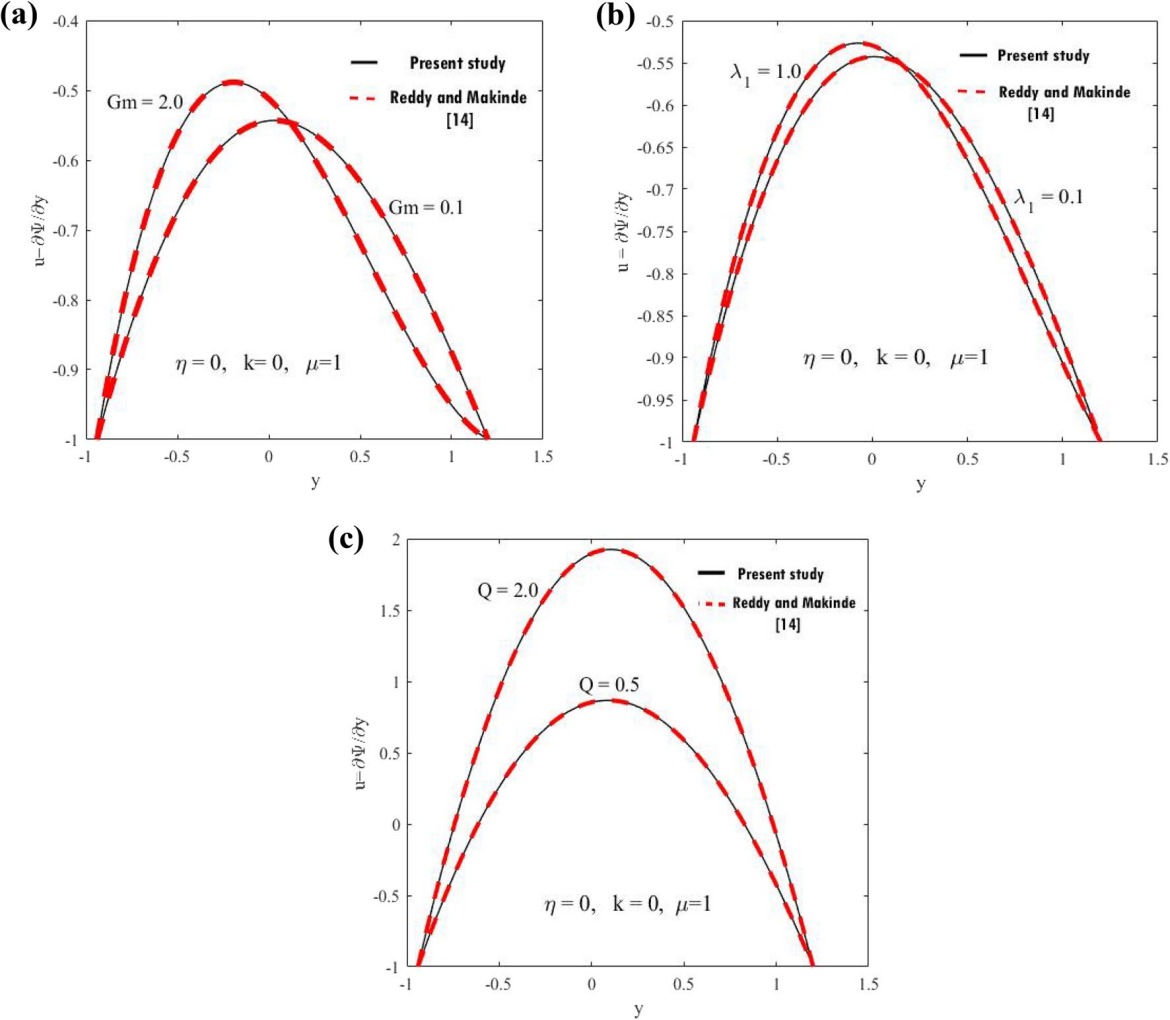
Comparison of velocity profiles. (**a**) For two values of $Gm.$ (**b**) For two values of $\lambda_{1} .$ (**c**) For two values of $Q.$

**Figure 10:**
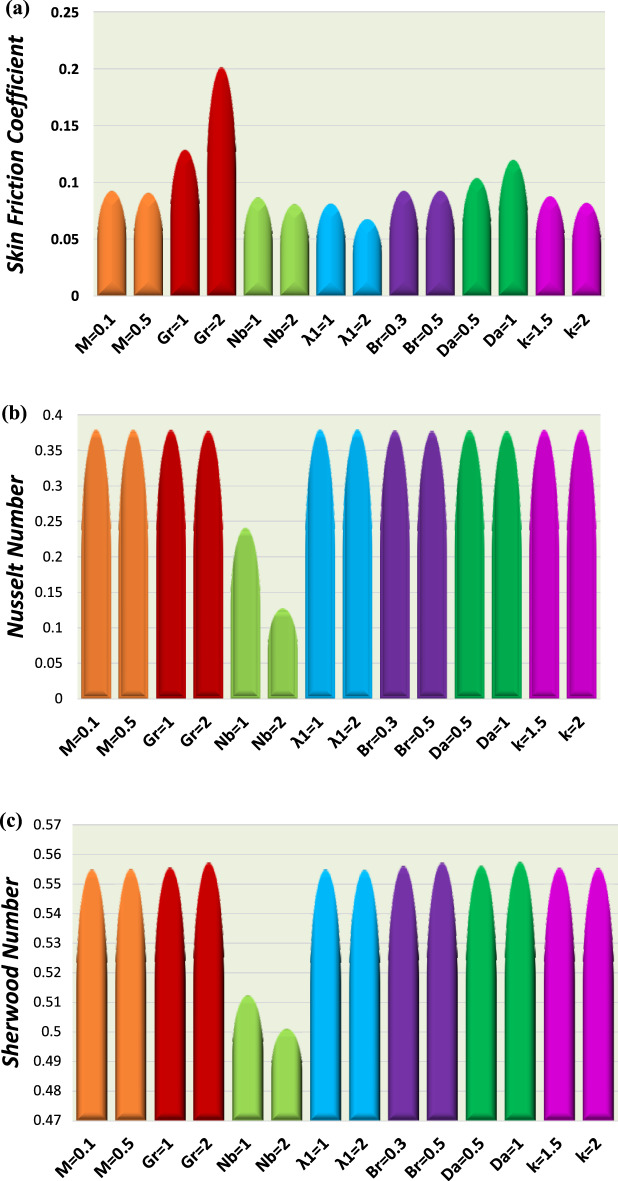
Discrepancy of the three quantities (**a**) the skin-friction coefficient. (**b**) Nusselt number. (**c**) Sherwood number.

**Figure 11:**
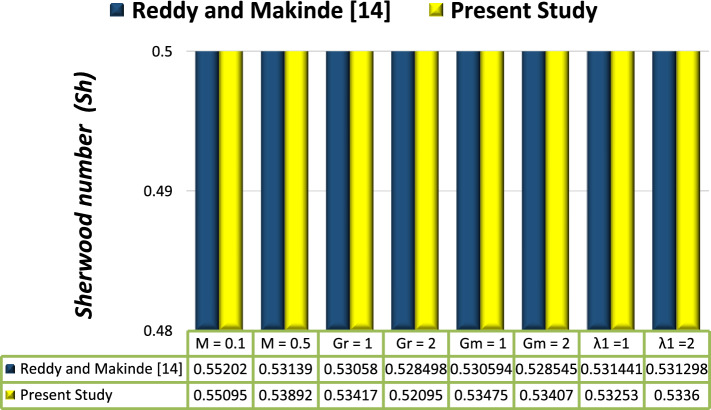
Comparison between previous work.

In the existing sector, the physical implication factors such as nanoparticle size between 1 and 100 nm that permits a longer circulation half-life in vivo and experience reduced hepatic filtration, Grashof number $$Gr$$$$\left( {0.5 \le Gr \le 2} \right),$$ nanoparticle Grashof number $$Gm$$ $$\left( {0.5 \le Gm \le 2} \right),$$ Hartmann number $$M$$$$\left( {0 \le M \le 1.5} \right),$$ Darcy number $$Da$$$$\left( {0.3 \le Da \le 0.9} \right),$$ thermophoresis parameter $$Nt$$$$\left( {0.2 \le Nt \le 0.8} \right),$$ Brownian motion parameter $$Nb$$$$\left( {0.2 \le Nb \le 0.8} \right),$$ Eckert number $$Ec$$$$\left( {0.1 \le Ec \le 0.7} \right),$$ phase difference $$\varphi$$$$\left( {\frac{\pi }{3} \le \varphi \le \frac{\pi }{6}} \right),$$ the relaxation to retardation time ratio of the upper channel $$\lambda_{`1}$$$$\left( {0.5 \le \lambda_{`1} \le 2} \right),$$ viscosity constant $$k$$$$\left( {1 \le k \le 2.5} \right),$$ flow rates $$Q$$ $$\left( {0.5 \le Q \le 2} \right),$$ Brinkman number $$Br$$ $$\left( {0.1 \le Br \le 1.3} \right),$$ Prandtl number $$\Pr$$ $$\left( {1 \le \Pr \le 2.5} \right),$$ width of the upper channel $$d_{1}$$$$\left( {0.3 \le d_{1} \le 0.6} \right),$$ and the viscosity constant $$\gamma_{1}$$$$\left( {0.5 \le \gamma_{1} \le 2} \right)$$ against velocity profiles, temperature, nanoparticle concentration, microrotation, pressure gradient, shear stress, heat flux, and nanoparticle volume flow are scrutinized through Figs. 2, 3, 4, 5, 6, 7, 8, 9, 10 and 11.

### Velocity distribution

According to Fig. 2, different values of $M,$ $Gr,$ $Gm,$ $Q,$ $Da,$ $k$ are displayed. There have been reports of parabolic velocity profiles. We also determined that $M$ does not influence velocity in the interval $- 1 \le y \le - 0.7,$ that it drops in the interval $- 0.7 \le y \le 0.44,$ and that it increases in the interval $0.44 \le y \le 1.2.$ When $Gr$ and $Gm,$ are increased, the velocity rises in the interval $- 1 \le y \le 0.2,$ falls in the interval $0.2 \le y \le 1.2,$ and rises across the full $y{ - }axis$ range when $Q$ is raised. Figure 2b, the cooling of the arterial walls ($Gr > 0$) has been considered. It is noticed that in the case of cooling of the arterial walls, velocity increases to the left of the channel, whereas an opposite behavior is observed near the arterial walls. It is also oscillatory, with the $Da$ falling in the interval $- 1 \le y \le - 0.7,$ rising in the interval $- 0.7 \le y \le 0.4,$ and falling again in the period $0.4 \le y \le 1.2.$ In contrast, increases in $k$ cause a decrease in velocity in the interval $- 1 \le y \le 0.1,$ and an increase in the period $0.1 \le y \le 1.2.$ It is also worth noting that the velocity profiles are also in compliance with boundary conditions, while nanoparticles are important to enrich or diminish the velocity rate. The nanoparticles phenomena distribution of the flow field is affected by three parameters, namely, the nanoparticle Grashof number, the Brownian parameter, and the thermophoresis parameter.

### Temperature distribution

Figure 3 depicts the temperature's fluctuation over $M,\,Br,\,Nt\,$ and $Nb$. A considerable increase in the temperature $\theta$ distribution is observed when the temperature rises with growth, which is consistent with the effective transfer of nanoparticles from the wall to the fluid. The magnetic field does not influence temperature. The temperature distribution is likewise self-evidently in compliance with the boundary conditions. These results are supported from the physical point of view and hold good with the results obtained by Reddy and Makinde^14^. From the observation of the results, it has been noted that the parameters involved have a similar role in the temperature, since the temperature determines the average kinetic energy which is related to the motion of fluid particles.

### Nanoparticle concentration distribution

Variations in $$\Pr ,$$$$Ec,$$$$Nt$$ and $$Nb$$ are shown in Fig. 4 about nanoparticle concentration fluctuations $$y - axis$$ concerning. If you increase $$\Pr ,$$$$Ec,$$$$Nt$$, you lower the nanoparticle concentration $$\Omega .$$ But if you increase $$Nb$$, you raise it. The advent of quicker random motion of the nanoparticles at higher numerical values of the Brownian motion parameter speeds up the diffusion process. Rising curves consequently demonstrate a rise in nanoparticle concentration. The hotter gold particles' quick transition from a hotter to a cooler area is also illustrated by this Brownian motion contribution. It is also evident that the distribution of concentrations meets all boundary conditions. This is in good agreement with what was obtained in clinical practice because the nutrients diffuse out of the blood vessels to neighboring tissues^35^.

### Microrotation distribution

By selecting four distinct values for $$M,$$ $$d_{1} ,$$ and $$\gamma_{1}$$, the microrotation fluctuations are obtainable in Fig. 5. By raising $$M,$$ microrotation velocity $$w$$ increases in the interval $$- 1 \le y \le - 0.55,$$ but decreases in the interval $$- 0.55 \le y \le 1.2.$$ With rising $$k$$ and $$d_{1}$$ in the interval $$- 1 \le y \le 0,$$ microrotation velocity decreases. Nevertheless, it increases in the interval $$0 \le y \le 1.2,$$ and it is rises with an increase $$\gamma_{1}$$ in the interval $$- 1 \le y \le 0.3,$$ however it decreases in the interval $$0.3 \le y \le 1.2.$$ If we consider that the Hartmann number viscosity constant which is a resistance to flow in the upper channel, and its magnitude is directly proportional to the microrotation velocity you can expect this impact. Considering that the Hartmann number, viscosity constant, and upper channel width all act as flow barriers whose sizes are proportional to the microrotation velocity, such an impact is not entirely surprising. Microrotation velocity oscillates, which may be due to peristalsis, as shown in Fig. 5. In addition, the boundary criteria are met by the micro rotational velocity. Otherwise, after $$y = - 0.3$$, it has an opposite behavior, i.e. the behavior of $$M,\,k,\,d_{1}$$ and $$\gamma_{1}$$ in the interval $$y$$ ∈ $$[ - 0.6, - 0.5]$$, $$[ - 0.02,0]$$, $$[0.03, - 0.8]$$ and $$[ - 0.03, - 0.7]$$ respectively, it has an opposite behavior, i.e. the behavior of $$w$$ in the above interval, is an inversed manner of its behavior in the interval $$y$$ ∈ $$[ - 5,\,1.2]$$, $$[0,\,1.2],$$$$[ - 0.8,\,1.2]$$ and $$[ - 0.7,\,1.2]$$ respectively.

### Pressure gradient distribution

For different values of $$M,$$$$Da,$$$$\varphi ,$$ the distributions of the pressure gradient $$\frac{dp}{{dx}}$$ throughout the distance $$x \in \,\,[0\,,\,1.5]$$ are revealed in Fig. 6 which shows that when $$M$$ grows, the pressure gradient reduces in the interval $$0 \le x \le 0.75,$$ increases in the interval $$0.75 \le y \le 1.45,$$ and subsequently decreases in the interval $$1.45 \le y \le 1.5.$$ It also increases in the interval $$0 \le x \le 0.7$$ with increasing $$\lambda_{1}$$ and $$Da$$, and decreases in the interval $$0.7 \le x \le 1.45$$ then decreases again after a short length of time in the interval $$1.45 \le y \le 1.5.$$ It declines with an increase of $$\varphi$$ in the entire range of $$x - axis.$$. These results reveal that to maintain the same flux throughout the channel's broadest region, a significantly larger pressure gradient is required. From these figures, we observe that a much large pressure gradient is required to maintain the same flux to pass it for the widest part of the channel $$x \in [0,\,1.5].$$ On the other hand, in a narrow part of the channel $$x \in [0,\,0.8],\,$$ whereas an opposite behavior is observed at $$x = 0.8.$$ This is well in agreement with the physical situation.

### Shear stress, heat flux, and nanoparticle volume flux

As portrayed in Fig. 7, The shear stress $C_{f} ,$ the heat flux $Nu$ and the nanoparticle volume flux $Sh$ for various parameter values have all been measured. The shear stress rises as $M$ rises in the interval $- 1 \le y \le 1,$ while declining in the interval $1 \le y \le 1.2.$ Shear stress rises and declines with the rise of the $Q,$ but the shear stress varies as it approaches unity in the interval $0.9 \le y \le 1.2$. However, while heat flux drops with rises $Nt$ in the interval $- 1 \le y \le 0.25,$ it rises in $0.25 \le y \le 1.2,$ as well it declines and rises with an increase of $Br,$ and there is a minor fluctuation on heat flux in the interval $0.7 \le y \le 0.85$, which approaches the unity. As $Nb$ rises, nanoparticle volume flux $Sh$ falls in the interval $- 1 \le y \le 0.1,$ while it grows in the interval $0.1 \le y \le 1.2$. In addition, nanoparticle volume flux falls with increasing $\Pr$ in the interval $- 1 \le y \le 0.8,$ whereas declines in the interval $0.8 \le y \le 1.2.$ as well.

Figure 8 illustrates how various parameter values affect the velocity $u$, temperature $\theta$ and microrotation velocity $w$. It is seen that the velocity grows with the growing of $Q$ and $Da$ at $\varphi \ne 0,\,\,\varphi = 0,$ although the values of the velocity at $\varphi = 0$ is greater than the values of velocity at $\varphi \ne 0,$ whereas, the temperature rises with growing of $Nb$ and it declines with growing of $Nt,$ while the values of the temperature at $\varphi \ne 0$ is greater than the values of temperature at $\varphi = 0,$ as well, the microrotation velocity declines with growing of $M$ and $\gamma_{1}$ at $\phi \ne 0,\phi = 0$ while the values of the microrotation velocity at $\varphi = 0$ is greater than the values of microrotation velocity at $\varphi \ne 0.$

Figure 9 shows the disparity of the velocity regarding $y{ - }axis$ two values of $Gr,\lambda_{1} \,$and $Q.$ It is observed that the present work coincides with the work made by Reddy and Makinde^14^ when $\mu = 1,\eta = 0,k = 0.$

Figures 10 and 11 display a bar chart view. A comparison between the numerical results of the current investigation and a previously published article by Reddy and Makinde^14^ is also provided.

Tables 1 and 2 also display estimations for the behavior of flow variables connected to various parameters, and validations with previous work. Results are in good accord with the findings in the graphs.

**Table 1:** Numerical values of the skin-friction coefficient, Nusselt number, and Sherwood number on the upper wall $l_{1}$ when *x* = 0.1.

*M*	*GR*	*Nb*	*λ*_1_	*Br*	*Da*	*k*	$C_{f}$	$Nu$	$Sh$
0.1	0.5	0.2	0.5	0.1	0.3	1	0.092497	0.379490	0.555089
0.5							0.091045	0.379440	0.555139
	1.0						0.128875	0.378922	0.555656
	2.0						0.201573	0.377201	0.557377
		1.0					0.086834	0.241195	0.512508
		2.0					0.080917	0.127695	0.501249
			1.0				0.081469	0.379536	0.555043
			2.0				0.067834	0.379602	0.554976
				0.3			0.092497	0.378345	0.556233
				0.5			0.092497	0.377201	0.557377
					0.5		0.103924	0.378253	0.556326
					1.0		0.119938	0.376933	0.557646
						1.5	0.087922	0.379010	0.555568
						2.0	0.082113	0.379126	0.555452

**Table 2:** Comparison between previous work on the upper wall $l_{1}$ when $x=0.3.$

				Reddy and makinde^14^	Present study
M	Gr	Gm	$\lambda$	$Sh$	$Sh$
0.1	0.5	0.5	0.5	0.55202	0.55095
0.5	0.5	0.5	0.5	0.53139	0.58892
0.1	1	0.5	0.5	0.53058	0.54417
0.1	2	0.5	0.5	0.528498	0.55095
0.1	0.5	1	0.5	0.530594	0.54475
0.1	0.5	2	0.5	0.528545	0.55407
0.1	0.5	0.5	1	0.531441	0.54253
0.1	0.5	0.5	2	0.531298	0.54360

Table 1 is erected to display the numerical values of skin-friction coefficient, Nusselt number, and Sherwood number for different parameters. It is realized that the Nusselt number drops and the Skin friction coefficient decline, while the Sherwood number rises for growing values of $M,$ while the skin-friction coefficient and Sherwood number rise with the growth of the $Gr,$ as well the Nusselt number decreases with the growth of the $Gr$ and $Nb.$ The Nusselt number rises with the growing of $\lambda_{1} .$ Therefore skin-friction coefficient, and Sherwood number decline with growing of $Nb,$ $\lambda_{1},$ and $k,$ as well there is no effect of $Br$ on the skin-friction coefficient, while Nusselt number declines and Sherwood number rises with growing of $Br$, as well the skin-friction coefficient and Sherwood number rise with the growing of $Da,$ while the Nusselt number declines with growing of $Da.$ The Nusselt number rises and the Sherwood number declines with increasing of $k.$

Table 3 indicates the maximum residual error (MRE) obtained during numerical computing process, which show the convergence and accuracy of the proposed method. Table 4 demonstrated the numerical computing values of mesh points estimated for variants of tolerance for each fluidic parameter by proposed scheme. The number of ODEs and BCs for numerically evaluated data involved in fluidic parameter are tabulated in Tables 5 and 6, respectively.

**Table 3:** Relative error magnitude of the fluid model.

Index-	Case-I	Case-II	Case-III	Case-IV
1	4.49426439593887e−13	6.15910035042290e−13	1.05787759940874e−12	1.13110084181839e−12
2	6.15910035042290e−13	6.68682885102556e−13	5.71938951406443e−13	5.01022618461845e−13
3	6.15910035042290e−13	6.75518211253230e−13	7.35720735747327e−13	7.97859562138294e−13
4	4.49426439593887e−13	6.15910035042290e−13	1.05787759940874e−12	1.13110084181839e−12
5	1.21213177775084e−13	2.29142788706109e−13	3.64931821737948e−13	5.25916022788075e−13
6	6.15910035042290e−13	1.53437572843295e−12	2.85768811925704e−12	4.64495166842510e−12
7	6.15910035042290e−13	6.01086989730102e−13	5.87780837205688e−13	2.80621421853545e−13
8	6.15910035042290e−13	5.33130348732800e−10	2.06260181321904e−12	1.38344553736849e−12
9	6.15910035042290e−13	1.71503502795156e−10	2.33438715364602e−10	6.76891366202345e−10
10	6.15910035042290e−13	2.15483119951417e−10	2.18895843740224e−12	2.08597516069485e−12
11	6.15910035042290e−13	5.67053177099433e−13	5.04979329232559e−13	4.32870251995460e−13
12	6.15910035042290e−13	2.29142788706109e−13	1.36851290035339e−13	1.00138259795431e−13
13	6.15910035042290e−13	8.75762439753214e−13	1.16793455868264e−12	1.49405598385667e−12
14	6.15910035042290e−13	2.34169821536561e−13	2.20806265269917e−13	2.28873639144344e−13

**Table 4:** Mesh points analysis of the fluid model.

Scenarios	Case-I	Case-II	Case-III	Case-IV
1	892	894	894	896
2	894	894	895	893
3	894	894	894	894
4	892	894	894	896
5	893	893	893	894
6	894	894	893	894
7	894	896	896	935
8	894	450	534	837
9	894	894	894	894
10	892	894	894	896
11	893	893	893	894
12	894	894	893	894
13	894	896	896	935
14	894	450	534	837

**Table 5:** Data from numerical simulation of ODEs for the fluid model.

Scenarios	Case-I	Case-II	Case-III	Case-IV
1	25,959	25,995	27,345	27,831
2	25,995	25,995	26,013	25,977
3	25,995	25,995	25,995	25,995
4	25,959	25,995	27,345	27,831
5	25,977	25,976	25,977	25,995
6	25,995	25,995	25,977	25,995
7	25,995	26,031	26,031	26,733
8	25,995	14,408	22,956	29,477
9	25,995	25,995	25,995	25,995
10	25,959	25,995	27,345	27,831
11	25,977	25,976	25,977	25,995
12	25,995	25,995	25,977	25,995
13	25,995	26,031	26,031	26,733
14	25,995	14,408	22,956	29,477

**Table 6:** Numerical data of BCs for the fluid model.

Scenarios	Case-I	Case-II	Case-III	Case-IV
1	77	77	77	77
2	77	77	77	77
3	77	77	77	77
4	77	77	77	77
5	77	77	77	77
6	77	77	77	77
7	77	77	77	77
8	77	74	107	108
9	77	74	74	74
10	77	74	107	107
11	77	77	77	77
12	77	77	77	77
13	77	77	77	77
14	77	77	77	77

## Conclusion

Peristaltic micropolar nanofluid flow in an asymmetric channel is the current focus of the research. An asymmetric channel was modeled using micropolar nanofluids. Use long-wavelength and low Reynolds number assumptions to simplify the non-dimensional governing equations of the flow, and the Rung–Kutta method to solve them numerically. According to the literature, the use of nanotechnology in medical science opens a new field of study for the beneficial effects of activation energy since nanoparticles help treat several disorders through peristaltic flow. The successful delivery of drugs or medical care to the damaged tissue or organ is made possible by such biological transport.

The following is a list of the main conclusions of the analysis that was done:

- The present study puts forward an important note that for peristaltic flow of a micropolar fluid with nanoparticles can be controlled by suitably adjusting the micropolar parameter, thermophoresis parameter, nanoparticle Grashof number, and Brownian motion parameter.
- In the center of the channel, velocity decreases, while its walls show the opposite tendency.
- For Gr & Gm, the fluid velocity profile increases near the upper channel.
- Increasing and decreasing micropolar rotation across the walls has been discovered to improve the Hartman number.
- The temperature profile rises with a rise in Nb, Nt and Br.
- The velocity distribution detected a reverse trend on the channel walls to micropolar nanofluid Darcy number.
- For the ordinary differential equations that arise in this paper, a numerical solution approach is offered.
- Researchers in science and engineering, as well as those working on the development of micropolar nanofluid mechanics, might find the findings in this paper useful.

## Future perceptions

The Lobatto IIIA scheme, Finite Difference method, Keller-box scheme and the Chebyshev spectral method may be implemented for the numerical treatment of various prospective applications appearing in bioinformatics, fluid mechanics problems, financial mathematics of vital significance^7,40^.

## Data Availability

The datasets used and/or analyzed during the current study available from the corresponding author on reasonable request.

## List of symbols

$a_{1} ,b_{1}$: Amplitudes of the wavy walls (m)
$$a,b$$: Amplitude ratios (m)
$d_{1} + d_{2}$: Width of the channel (m)
$d$: Mean half-width of the channel (m)
$\tilde{\chi }_{1} + \tilde{\chi }_{2}$: The geometry of the upper and lower surface
$\mu$: Viscosity constant of the nanofluid dynamics (kg/m s)
$$\lambda$$: Wavelength (m)
$$c$$: Wave speed (m/s)
$$\varphi$$: Phase difference
$\lambda_{1}$: The ratio of relaxation to retardation times
$$\lambda_{2}$$: Retardation time
$$\overline{{\mathop \gamma \limits^{.} }}$$: Shear rate
$\rho_{f}$: The density of the nanofluid (kg/m^3^)
$\rho_{p}$: The density of the fluid at a pressure constant (kg/m^3^)
$\rho_{c}$: The density of the fluid at constant velocity
$k,\gamma_{1}$: Viscosity constants for micropolar fluid
$\overline{w}$: The microrotation velocity components in the direction normal to both the $\overline{x}$ and $\overline{y}$ axes
$\overline{J}$: Micro-inertia constant
$$\alpha$$: Thermal diffusivity
$c_{f}$: Specific heat of nanofluid
$\tau$: The ratio of effective heat capacity of the nanoparticle material to the heat capacity of the fluid
$D_{B}$: The Brownian motion coefficient
$D_{T}$: The thermophoretic diffusion coefficient
$$T_{m}$$: Mean temperature
$$\upsilon$$: Kinematic viscosity (m^2/^s)
$c_{p}$: Specific heat at constant pressure (J/kg/k)
$q$: The flux of the nanofluid
$\psi \,^{\prime}$: The stream function
$\tilde{\xi },\tilde{\zeta }$: Velocity components in the fixed frame (m/s)
$\overline{t}$: Time in the fixed frame (s)
$\overline{P}$: Pressure in the fixed frame (Pa) Pa = (kg/ms^2^)
$$\theta$$: Temperature distribution
$\Omega$: Nanoparticles concentration distribution
$g$: Acceleration due to gravity (m/s)
$\varsigma_{t}$: Coefficient of thermal expansion
$\varsigma_{c}$: Coefficient of viscosity at constant concentration
$T_{1} ,T_{o}$: The temperature of the lower and upper walls of the channel (K)
$C_{1} ,C_{o}$: The concentration of the lower and upper walls of the channel (kg/m^3^)
$T$: The temperature of the fluid (K)
$C$: The concentration of the fluid (kg/m^3^)
$\sigma$: Electrical conductivity of the fluid
$B_{0}$: The intensity of the external magnetic field (Wb/m^2^)
$$k_{1}$$: The permeability (W/m/K)
$$\eta$$: The porosity
$$\delta$$: Wavenumber
$$M$$: Hartmann number
$${\text{Re}}$$: Reynolds number
$$\Pr$$: Prandtl number
$$Gr$$: Grashof number
$Gm$: Nanoparticle Grashof number
$$Br$$: Brinkman number
$$Nt$$: Thermophoresis parameter
$Nb$: Brownian motion parameter
$$Da$$: Darcy number
$$Ec$$: Eckert number
